# Grain setting defect1 (GSD1) function in rice depends on *S*-acylation and interacts with actin 1 (OsACT1) at its C-terminal

**DOI:** 10.3389/fpls.2015.00804

**Published:** 2015-10-01

**Authors:** Jinshan Gui, Shuai Zheng, Junhui Shen, Laigeng Li

**Affiliations:** National Key Laboratory of Plant Molecular Genetics, Institute of Plant Physiology and Ecology, Shanghai Institutes for Biological Sciences, Chinese Academy of SciencesShanghai, China

**Keywords:** remorin, *S*-acylation, plasma membrane, plasmodesmata, rice

## Abstract

Grain setting defect1 (GSD1), a plant-specific remorin protein specifically localized at the plasma membrane (PM) and plasmodesmata of phloem companion cells, affects grain setting in rice through regulating the transport of photoassimilates. Here, we show new evidence demonstrating that GSD1 is localized at the cytoplasmic face of the PM and a stretch of 45 amino acid residues at its C-terminal is required for its localization. Association with the PM is mediated by *S*-acylation of cysteine residues Cys-524 and Cys-527, in a sequence of 45 amino acid residues essential for GSD1 function in rice. Furthermore, the coiled-coil domain in GSD1 is necessary for sufficient interaction with OsACT1. Together, these results reveal that GSD1 attaches to the PM through *S*-acylation and interacts with OsACT1 through its coiled-coil domain structure to regulate plasmodesmata conductance for photoassimilate transport in rice.

## Introduction

A variety of proteins are associated with the plasma membrane (PM) to form PM-associated platforms for carrying out complex biological processes (Astro and de Curtis, 2015). Several means including transmembrane domain clasps, protein-protein interactions, lipid binding motifs/domains or lipidation linkages assist proteins to associate with the membrane. Remorins are considered PM microdomain proteins and play various roles in plants (Mongrand et al., 2004; Lefebvre et al., 2007, 2010; Raffaele et al., 2009; Jarsch and Ott, 2011; Jarsch et al., 2014). The remorin protein family in each plant species includes a large number of members belonging to six groups (Raffaele et al., 2007). Usually remorins contain variable N-terminal regions and conserved domain structures in their C-terminal sequence. Recent studies indicate that post-translational lipidation is required for the membrane association of remorin proteins. Membrane association is mediated through *S*-acylation but the *S*-acylation patterns differ between remorin family members or groups (Konrad et al., 2014). Some of the *Arabidopsis* remorins are associated with the PM possibly through *S*-acylation at different sequence positions but this association does not determine their memebrane microdomain localization (Konrad et al., 2014). While *S*-acylation of remorins plays a key role in affecting the performance of various remorin functions, how different pattern of remorin *S*-acylation is related to remorin function remains to be investigated.

*S*-acylation (also known as palmitoylation) is a reversible post-translational modification of covalently linking a 16-carbon palmitate or 18-carbone stearate through a thioester bond to cysteine residues of proteins (Greaves and Chamberlain, 2007; Linder and Deschenes, 2007; Sorek et al., 2007). *S*-acylation has been widely investigated in mammalian cells but rarely studied in plants. As *S*-acylation is reversible, the cycle of acylation and deacylation of cysteine may serve as a dynamic regulatory mechanism for shuttling proteins between the cytosol and the membrane, thereby regulating their localization and function (Greaves and Chamberlain, 2007; Linder and Deschenes, 2007; Sorek et al., 2007). A number of S-acylated PM proteins, such as small GTPase RACs (AtROP6, AtRAC7, AtRAC8, and AtRAC10) (Lavy et al., 2002; Sorek et al., 2007, 2010), heterotrimeric guanine nucleotide-binding proteins (G proteins) GPA1 and AGG2 (Adjobo-Hermans et al., 2006; Zeng et al., 2007), calcium signaling proteins OSCPK2 and CBL1 (Martin and Busconi, 2000; Batistic et al., 2008), and protein phosphatase type 2C (PP2C) proteins POLTERGEIST (POL) and PLL1 (Gagne and Clark, 2010), have been identified in plants. In *Arabidopsis*, proteomic studies identified 581 putatively S-acylated proteins including two remorins AtREM1.2 (At3g61260) and AtREM1.3 (At2g45820) (Hemsley et al., 2013). Recently, *S*-acylation of *Arabidopsis* remorin AtREM6.4 (At4g36970) and Medicago SYMREM1 were examined in tobacco (Konrad et al., 2014). However, since no consensus motifs have been identified for protein *S*-acylation, the prediction of *S*-acylation is extremely difficult (Roth et al., 2006; Hemsley et al., 2013). Identifying and understanding the sites and significance of *S*-acylation in the performance of protein function is a critical question to be answered.

In a previous study, we identified a remorin gene, *grain setting defect1* (*GSD1*), which affects grain setting in rice through regulating plasmodesmatal (PD) conductance (Gui et al., 2014). *GSD1*, which belongs to remorin group 6, encodes a protein which consists of a variable N-terminal region and conserved domains at the C-terminal region. *GSD1* is localized on the PM and plasmodesmata and is specifically expressed in phloem companion cells. Previous results suggest that GSD1 plays a role in modulating PD aperture by connecting the PM to OsACT1, which is a part of the desmotubule structure in the PD channels of companion cells. However, how GSD1 is localized on the PM and how GSD1 interacts with OsACT1 in the desmotubule structure is unknown.

In this paper, we present new evidence to demonstrate that *S*-acylation at the GSD1 C-terminal region mediates PM association and a new actin-binding domain containing a coil-coiled domain structure is responsible for its interaction with OsACT1. Such a GSD1 structure is essential for performance of the GSD1 function in rice.

## Materials and Methods

### Plant Material

Wild-type rice (*Oryza sativa*) used in this study was *O. sativa japonica* cv Zhonghua 11. Rice plants were grown in a phytotron under conditions of approximately 60% humidity, 12/12 h light/dark cycles, 28°C constant temperature regime, and photon flux density at 200 to 250 μmol m^-2^ s^-1^. The transient expression of GSD1 was performed using tobacco *Nicotiana benthamiana* which was grown in a phytotron under conditions of approximately 70% humidity, 16/8 h light/dark cycles, 22/20°C day/night temperature regimes.

### Generation of Constructs

To examine subcellular localization, the full length *OsREM4.1* (Os07g38170), *AtPDLP1* (At5g43980), *AtSAR1* (At1g56330), and mutated *AtSAR1* (Sar1H74L) cDNA were amplified by PCR. The PCR product was digested and subcloned into vector pCAMBIA1300-35S-GFP or pCAMBIA1300-35S-mCherry in fusion with a fluorescence protein to yield *GFP-OsREM4.1, PDLP1-GFP, PDLP1-mCherry, Sar1-mCherry* and *Sar1H74L-mCherry*, respectively.

To identify the GSD1 section responsible for PM association, a series of truncated GSD1 mutations were generated. The truncated *GSD1* fragments (*GSD1N, GSD1C1, GSD1C2, GSD1C3, GSD1C4, GSD1C5* and *GSD1C6*, see **Figure 2**) were fused to *GFP* C-terminal in *pCAMBIA1300-35S-GFP* vector. The truncated *GSD1* fragments were also fused to *YFP* N-terminal *YN* fragment and C-terminal *YC* fragment for BiFC. These constructs were named *YC-GSD1N, YC-GSD1C1* and *YC-GSD1C2*, respectively.

Site-specific mutations of cysteine to serine or alanine at *GSD1* C-terminal were introduced through PCR mutagenesis. Five cysteine mutations were generated and constructed into *pCAMBIA1300-35S-GFP*, yielding constructs named *GFP-M1, GFP-M2, GFP-M3, GFP-M4, GFP-M5, GFP-M6, GFP-M7, GFP-M8*, and *GFP-M9*, respectively.

For Co-IP analysis, *Myc*-tagged *GSD1N, GSD1C1* and *Myc*-tagged *GSD1C2* were generated.

To investigate GSD1 function performance in rice, the truncated or site-mutated *GSD1* formats were constructed into *pHB* vector for rice transformation. The accuracy of above constructs was confirmed by sequencing and the specific primers used in this study are shown in Supplemental Table S1.

### Transient Expression in Protoplast or in Tobacco Leaf

For transient expression, protoplasts were isolated from rice (*O. sativa japonica*) seedlings and tobacco leaf cells as previously described (Yoo et al., 2007). For PEG-calcium transfection, plasmid DNAs (about 1 μg/μL of total 40 μg DNA of each construct) were mixed gently with 100 μL of suspended protoplasts. Then, 140 μL PEG solution (40% PEG4000, 200 mM mannitol, and 100 mM CaCl_2_) was added to DNA and protoplasts mixture with gentle shaking. The mixture was incubated at 23°C for 15 min. After incubation, 1.5 mL of W5 solution [2 mM MES (pH 5.7), 154 mM NaCl, 125 mM CaCl_2_, and 5 mM KCl] was added and mixed gently with PEG4000. Protoplasts were collected by centrifugation at 100 *g* for 1 min and resuspended in 2 mL of W5 solution, then cultured in dark overnight at 23°C before visualized by a confocal laser scanning microscopy.

For transient expression in tobacco leaf, the construct was transformed into *Agrobacterium tumefaciens* GV3101. Transformed clones were grown overnight at 28°C in YEB medium (5 g/L beef extract, 1 g/L yeast extract, 5 g/L peptone, 5 g/L sucrose, and 0.5 g/L MgCl_2_) supplemented with 50 μg/ml kanamycin and 25 μg/ml rifampcin to stationary phase. Bacteria were sedimented by centrifugation at 5000 *g* for 5 min at room temperature and resuspended in injection solution [10 mM MgCl_2_, 10 mM MES (pH 5.7), and 20 μM acetosyringone] with an optical density (OD600) of 1.0. The *Agrobacterium* cells were infiltrated into tobacco abaxial leaf tissue using a syringe barrel. The infiltrated leaf was cultured for 48–72 h before being visualized under a confocal laser scanning microscopy (Zeiss LSM510). GFP was excited at 488 nm and captured at 505 to 555 nm. mCherry was excited at 587 nm and captured at 590–630 nm. YFP was excited at 512 nm and captured at 525–550 nm.

### Protease Sensitivity Assay

To examine GSD1 orientation on PM, protease sensitivity assay was carried out using transient expression GSD1 in tobacco leaf. After 2 days of incubation, the *Agrobacterium*-infiltrated tobacco leaf was cut and washed with PBS and incubated at 37°C with either 10 μg/mL proteinase K in PBS, 10 μg/mL proteinase K plus 0.1% Triton X-100 (v/v) in PBS, 0.1% Triton X-100 in PBS only, or PBS only for 30 min. After proteinase K incubation, the leaf was washed with PBS and ground in liquid nitrogen to fine powder. The sample was extracted with appropriate volume of SDS protein extraction buffer [0.5 M Tris-Cl, pH 6.8, 4.4% (w/v) SDS, 2% β-mercaptoethanol, 20% glycerol, and 0.036% bromophenol blue]. After being boiled for 10 min, the extracted proteins were separated on 12% SDS-PAGE and blotted onto a nylon membrane. Immunoblotting analysis was performed with rabbit polyclonal anti-GFP antibody.

### Secretory Pathway Analysis

To analyze the secretory pathway for GSD1 traffic to PM, Brefeldin A (BFA) and 2-bromopalmitate (2-BP) were used to treat the GSD1-expressed tobacco leaves. *GFP-GSD1* was co-infiltrated into the abaxial leaf sides with the *PDLP1-GFP* or Golgi marker *GmMan1-mCherry*. After incubation for 2 days, the leaf sectors were re-infiltrated with or without 50 μg/mL BFA and examined after 12 h incubation. For 2-BP assay, *GFP-GSD1* were infiltrated with or without 100 μM 2-BP and examined after 2 days incubation.

### Biotin Switch Assay of *S*-acylation

The biotin switch assay was performed as previously described (Hemsley et al., 2008) with minor modifications. Briefly, about 10 mg proteins of membrane fractions were solubilized and incubated with 25 mM *N*-ethylmaleimide (Thermo scientific) to mask free cysteines. Free *N*-ethylmaleimide was removed by methanol-chloroform precipitation. Precipitated proteins were solubilized and incubated with 1 M hydroxylamine (NH_2_OH) (Thermo scientific) to cleave a thioester bond and with 1 mM biotin-HPDP (Thermo scientific) to label new free cysteines. Hydroxylamine was replaced by Tris-HCl buffer as a control. Biotinylated proteins were then purified with 20 uL NeutrAvidin-agarose (Thermo Scientific) and analyzed by Western blotting using GSD1 specific antibodies.

### Analysis of Membrane Fractions

Membrane fractions were isolated from rice booting panicles (about 7 days before flowering). Equal volumes of 2-BP treated or not treated membrane fractions were further treated with Triton X-100 buffer [50 mM Tris-Cl, pH 7.4, 150 mM NaCl, 5 mM MgCl_2_, 1 mM EDTA, 1% Triton X-100, and 0.1% protease inhibitor cocktail (Promega)] at 4°C for 30 min. Then, Triton X-100 treated membrane fractions was separated by sucrose density gradients as described previously (Raffaele et al., 2009). Treated membrane fractions were mixed with sucrose to a final concentration of 52% sucrose (w/w) and loaded into a centrifuge tube. The load fraction was overlaid with 2.5 ml each of 40, 35, and 30% sucrose in TBS buffer (w/w), and then centrifuged at 200,000 *g* at 4°C for 16 h. The centrifuged solution was collected equally into 12 fractions and precipitated. Equal volumes of each fraction were analyzed by Western blotting.

### Phloem Loading Measurement

To analyze the rate of photoassimilate loading into phloem system, ^13^CO_2_ stable isotope was used to label photoassimilates according to established methods (Gui et al., 2014). After 12 h of photosynthesis fed with ^13^CO_2_, the rice plants were placed to dark conditions. Leaf blade, leaf sheath, and phloem exudate were collected after 6 h in dark, respectively, for soluble sugar determination.

## Results

### C-terminal Sequence is Involved in GSD1 Association with PM

In a previous study, we found that a remorin protein GSD1 in rice is localized on the PM (Gui et al., 2014). However, since GSD1 is overall hydrophilic in character and does not contain typical transmembrane structure, we investigated how it was localized on the PM. First, we established protoplast systems using rice and tobacco leaves to study GSD1 subcellular localization. GFP-tagged *GSD1* was expressed in rice and tobacco leaves and results showed that GSD1 predominantly resides in the membrane fraction and is specifically associated with the PM in both rice and tobacco protoplasts (Supplementary Figures S1A,B). This confirmed the feasibility of the tobacco protoplast system for the localization analysis.

To examine whether GSD1 resides inside or outside of the PM surface, GFP-GSD1 was expressed in tobacco leaf epidermis cells and treated with proteinase K in the presence or absence of detergent Triton X-100. Actin without detectable extracellular domain was used as a control. Neither GFP-GSD1 nor actin showed a detectable change in protein mobility or amount after proteinase K treatment (Supplementary Figure S1C). In contrast, proteinase K treatment in the presence of triton X-100 resulted in the disappearance of GFP-GSD1 and actin (Supplementary Figure S1C). This revealed that GSD1 is not sensitive to extracellular proteinase K treatment, indicating that GSD1 associates with PM at the cytoplasmic face.

**FIGURE 1:**
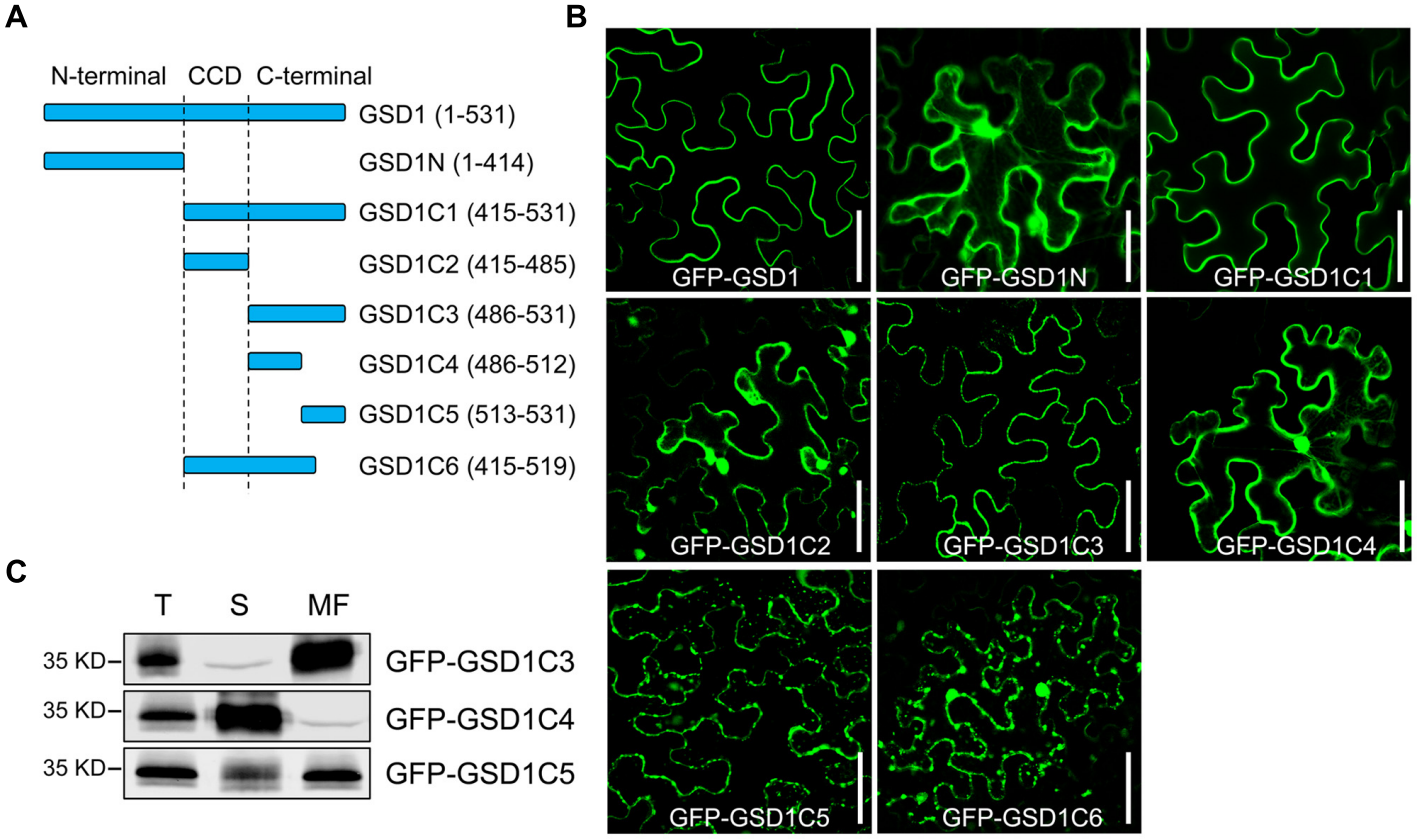
**A stretch of 45 amino acid residues at the C-terminal is sufficient for grain setting defect 1 (GSD1) association with PM. (A)** Schematic representation of full-length and truncated GSD1 fragments. CCD, coiled-coil domain. **(B)** Full-length and truncated GSD1 fragments were fused with GFP and expressed in tobacco leaves. Images show that GSD1C1 and GSD1C3 are localized specifically on PM. Bars = 50 μm. **(C)** Western blot detection of GFP-GSD1C3, GFP-GSD1C4, and GFP-GSD1C5 in soluble and membrane fractions of the transformed tobacco leaves. T, total; S, soluble; MF, membrane fractions.

The C-terminal region contains a predicted coiled-coil domain and a variable C-terminus tail. The N-terminal region is predicted to contain a putative GSK3 phosphorylation recognition site, a predicted Tyr-based sorting signal, a putative MAPK interacting motif, which may be involved in specific interactions in MAPK cascades or could be responsible for interactions within the protein complex (Supplementary Figure S2A). To identify which part of GSD1 is responsible for its PM localization, we generated a series of truncated GSD1 proteins (N-terminal part of GSD1: GSD1N, various C-terminal sections of GSD1: GSD1C1, GSD1C2, GSD1C3, GSD1C4, GSD1C5, and GSD1C6, **Figure 1A**), which were fused with a GFP at the N terminus and used for the examination of their cellular localization. The N-terminal part of GSD1, GSD1N, was localized in the cytoplasm, while GSD1C1, the part deleted with N-terminal, was associated with the PM, at the same location as the full-length GSD1 protein (**Figure 1B**). This result indicates that the GSD1 PM anchoring sequence is in its C-terminal. To map the exact PM anchoring sequence, further deletion analysis of a series of GSD1 truncated proteins was carried out. When GSD1C1 was cut into GSD1C2 and GSD1C3, GSD1C2 was localized in the cytoplasm, whereas GSD1C3 was exclusively associated with the PM (**Figure 1B**). As GSD1C3 was further divided into GSD1C4 and GSD1C5, GSD1C4 was present in cytoplasm, while GSD1C5 was predominantly localized on PM but also displayed weak signals in cytoplasm (**Figure 1B**). However, GSD1C6, with a stretch of 12 amino acid residues removed from the GSD1C1 end, was localized in the cytoplasm (**Figure 1B**). Taken together, these results demonstrate that a stretch of 12 amino acid residues is necessary but potentially not sufficient for GSD1 to be specifically associated with the PM. In addition to the 12 amino acid residues, other adjacent sequence may be needed to secure GSD1 on PM. Therefore, the distribution of GSD1C3, GSD1C4, and GSD1C5 in the cytoplasm and membrane was examined using plasmolysis and immunoblotting analysis. After plasmolysis, GSD1C3 showed a clear PM localization, while GSD1C4 displayed a typical cytoplasmic distribution (Supplementary Figure S3). GSD1C5 was predominantly localized on the PM, but also exist in cytoplasm (Supplementary Figure S3). Protein immunoblotting analysis using anti-GFP monoclonal antibodies indicated that GFP-GSD1C3 was predominantly localized in the membrane fraction, whereas GFP-GSD1C4 was localized in the soluble fraction (**Figure 1C**). GFP-GSD1C5 was predominantly present in the membrane fraction, but also present in small amounts in the soluble fraction (**Figure 1C**). Collectively, GSD1 truncation, plasmolysis and immunoblotting analyses revealed that GSD1C3, essentially a stretch of 45 amino acid residues, which is not conserved among remorins, is sufficient for GSD1 to anchor on the PM.

### *S*-acylation of C-terminal Sequence Responsible for Localization of GSD1 on the PM

**FIGURE 2:**
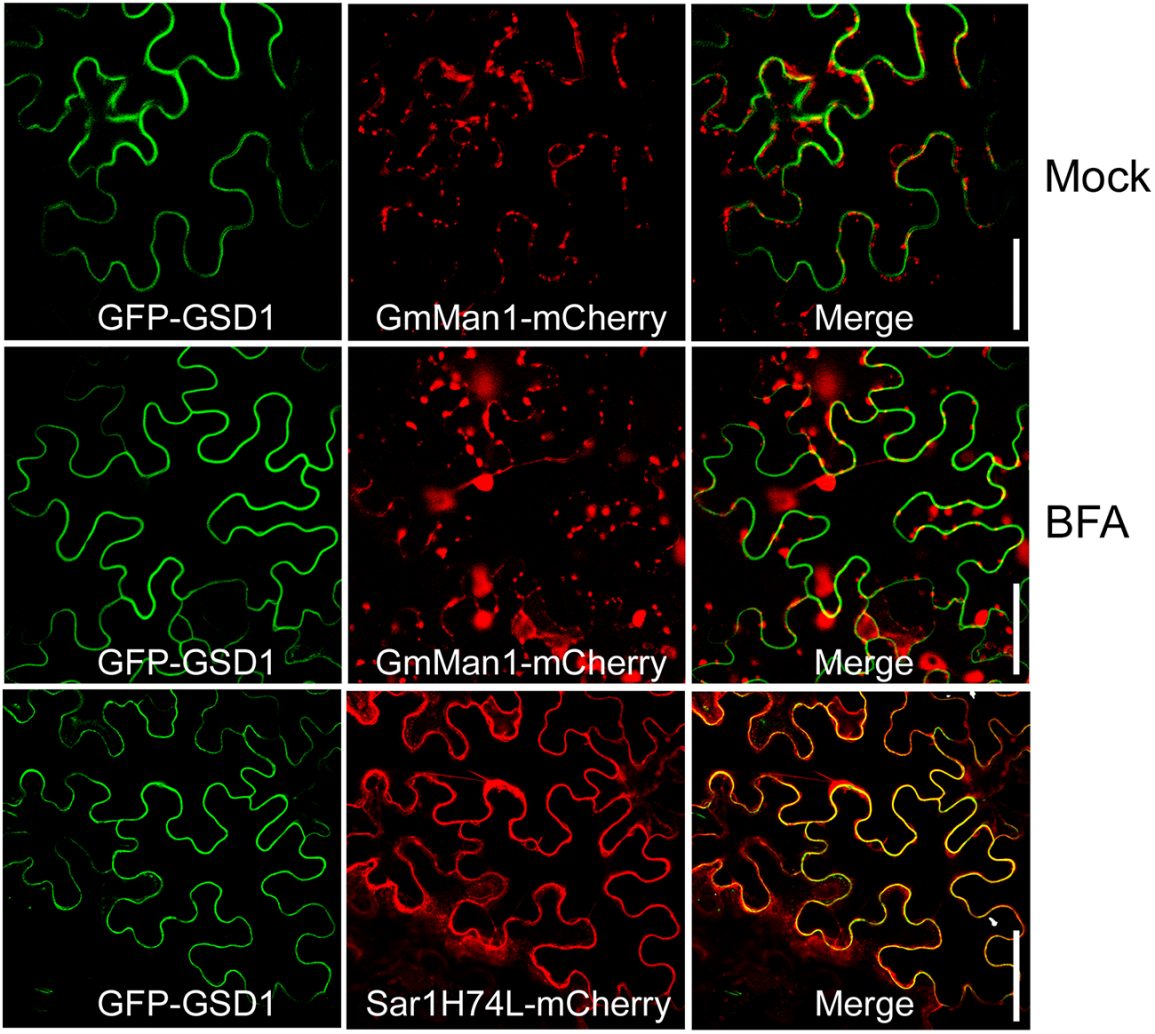
**Analysis of GSD1 association with PM.** Coexpression of GFP-GSD1 with Golgi marker GmMan1:mCherry or COPII-mediated ER-Golgi transport regulator mutant Sar1H74L-mCherry in tobacco leaves. Images show that GSD1 trafficking to PM is unaffected by BFA treatment, while subcellular localization of GmMan1:mCherry is changed. Additionally, GSD1 trafficking to PM are not affected when coexpressed with mutant Sar1H74L-mCherry. Bars = 50 μm.

ER-Golgi trafficking is a general pathway for protein delivery to the PM. To understand how GSD1 trafficking to the cytoplasmic face of the PM, Brefeldin A (BFA), an inhibitor for the ER-Golgi trafficking pathway and Sar1(H74L), a GTPase-defective mutant which inhibits the COPII-mediated ER-to-Golgi transport of several proteins, such as PDLP1, to the PM (Nebenfuhr et al., 2002; Thomas et al., 2008), were used to investigate the GSD1 trafficking. Coexpression of GmMan1:mCherry or PDLP1:mCherry with GFP-GSD1 in tobacco leaf showed that GSD1 localization to PM was unaffected by BFA treatment, while both GmMan1:mCherry and PDLP1:mCherry subcellular localization were strongly affected by BFA (**Figure 2**, Supplementary Figure S4). In addition, when wild-type Sar1-mCherry or mutant Sar1H74L-mCherry was coexpressed with GFP-GSD1, results showed that the GSD1 PM association was not affected (**Figure 2**, Supplementary Figure S4). These results demonstrated that the GSD1 localization to PM, which is insensitive to BFA treatment and Sar1H74L mutant, is independent to the ER-Golgi trafficking pathway.

**FIGURE 3:**
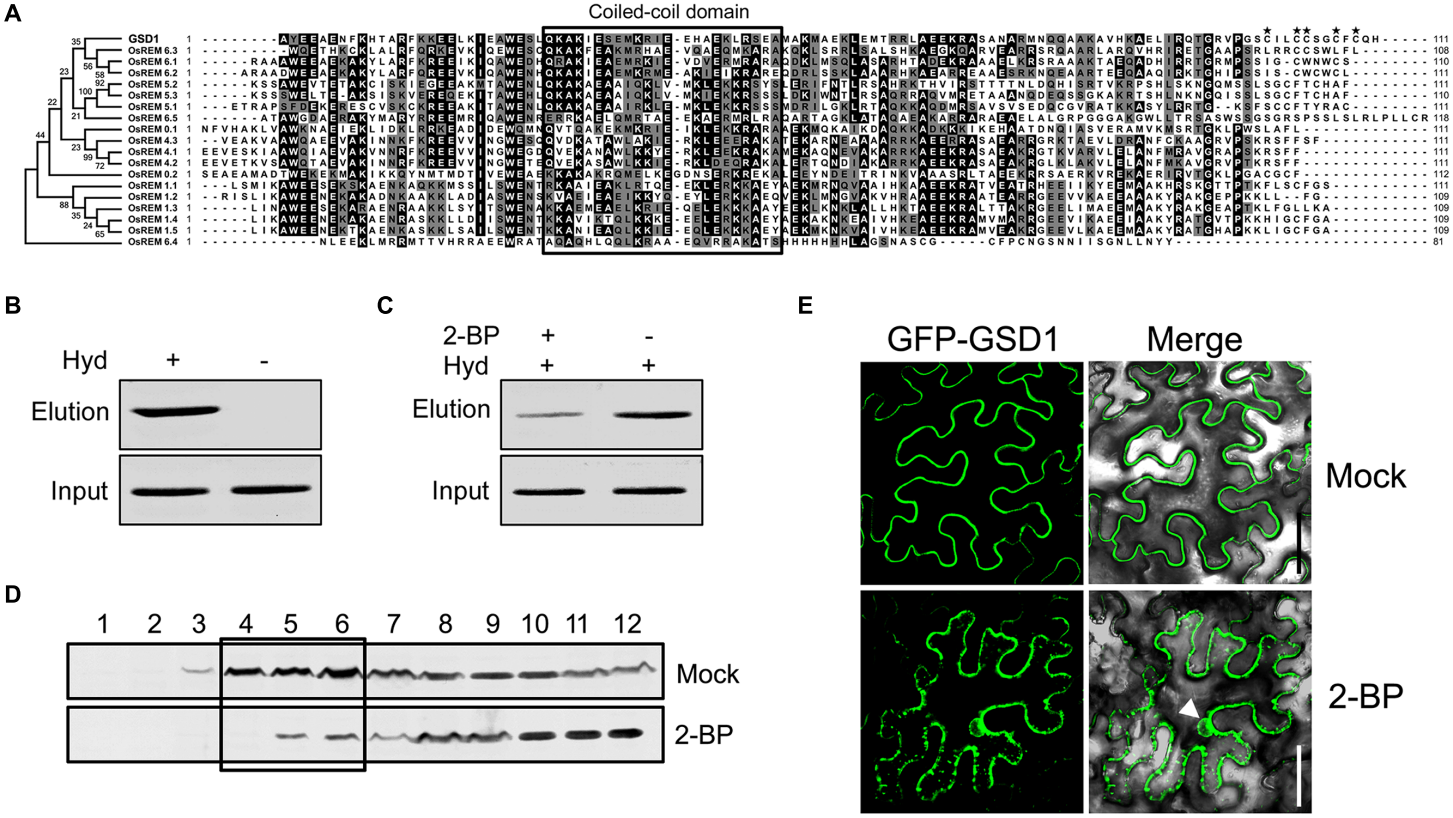
**Grain setting defect 1 association with PM through *S*-acylation. (A)** Alignment of the GSD1 C-terminal sequences with its homologous proteins. The C-terminal sequences contain a coiled-coil domain but vary at the end amino acid residues. Asterisk indicates possibly S-acylated cysteine residues. **(B)** Biotin switch assays of *S*-acylation state of GSD1 in rice membrane fractions. S-acylated GSD1 is detected by Western blotting in the elution fraction of the hydroxylamine-treated sample but not detected in the untreated sample. Hyd+, with hydroxylamine; Hyd-, without hydroxylamine. **(C)** Comparison of the GSD1 *S*-acylation with or without 2-bromopalmitate (2-BP) (an *S*-acylation inhibitor) treatment. 2-bromopalmitate treatment substantially reduced the amount of S-acylated GSD1. **(D)** Western blotting of GSD1 in sucrose density gradient fractions of membrane proteins from rice with GSD1 specific antibodies. GSD1 is predominantly detected in the upper fractions (fractions: 4–6), whereas 2-BP treatment substantially reduces GSD1 in these fractions. **(E)** Confocal microscopic observation of the subcellular localization of GFP-GSD1 in tobacco leaves under 2-BP treatment or mock solution. Arrowhead denotes GFP fluorescent in nucleus. Bars = 50 μm.

On the other hand, as the C-terminal sequence of the 45 amino acid residues in GSD1 is sufficient to mediate anchoring to the PM, we performed a comparative analysis of the C-terminal amino acid sequences with GSD1 homologous remorins in rice. As shown in **Figure 3A**, the C-terminal sequences were conserved in coiled-coil domain and adjacent sequences, but not conserved in the 12 amino acid residues which are essential for GSD1 to associate with the PM. Although the entire sequence of GSD1 protein is extremely hydrophilic, the 12 C-terminal amino acid residues are hydrophobic containing five cysteine residues which are predicted to be likely S-acylated (Supplementary Figures S5A,B) (Kyte and Doolittle, 1982; Ren et al., 2008). Thus, we examined whether GSD1 is S-acylated through the cysteine residues using a biotin switch assay in combination with a point mutation assay, which allows for the analysis of protein *S*-acylation and identification of *S*-acylation sites (Hemsley et al., 2008). GSD1 was isolated from rice tissue and analyzed for its *S*-acylation. As shown in **Figure 3B**, GSD1 was detected to be S-acylated in rice. When *S*-acylation was inhibited, the level of the S-acylated GSD1 was reduced substantially (**Figure 3C**). These results indicate that GSD1 is an S-acylated protein in rice.

When GSD1 protein from rice was fractionated using a sucrose density gradient (Raffaele et al., 2009), GSD1 was found to be present mainly in the upper fractions, which were often named the PM and membrane microdomains fractions (**Figure 3D**). When *S*-acylation was inhibited by 2-bromopalmitate, its proportion in each sucrose gradient fractions was substantially changed (**Figure 3D**).

We also investigated whether *S*-acylation affected the localization of GSD1 on the PM. When tobacco leaf cells expressing the GSD1 protein were treated with 2-bromopalmitate (2-BP), an *S*-acylation inhibitor, GSD1 was localized at both PM and cytoplasm. The loss of its specific localization on PM indicates that the mislocalization of GSD1 is caused by the inhibition of GSD1 *S*-acylation (**Figure 3E**).

### *S*-acylation Occurs at Cys-524 and Cys-527 of GSD1

**FIGURE 4:**
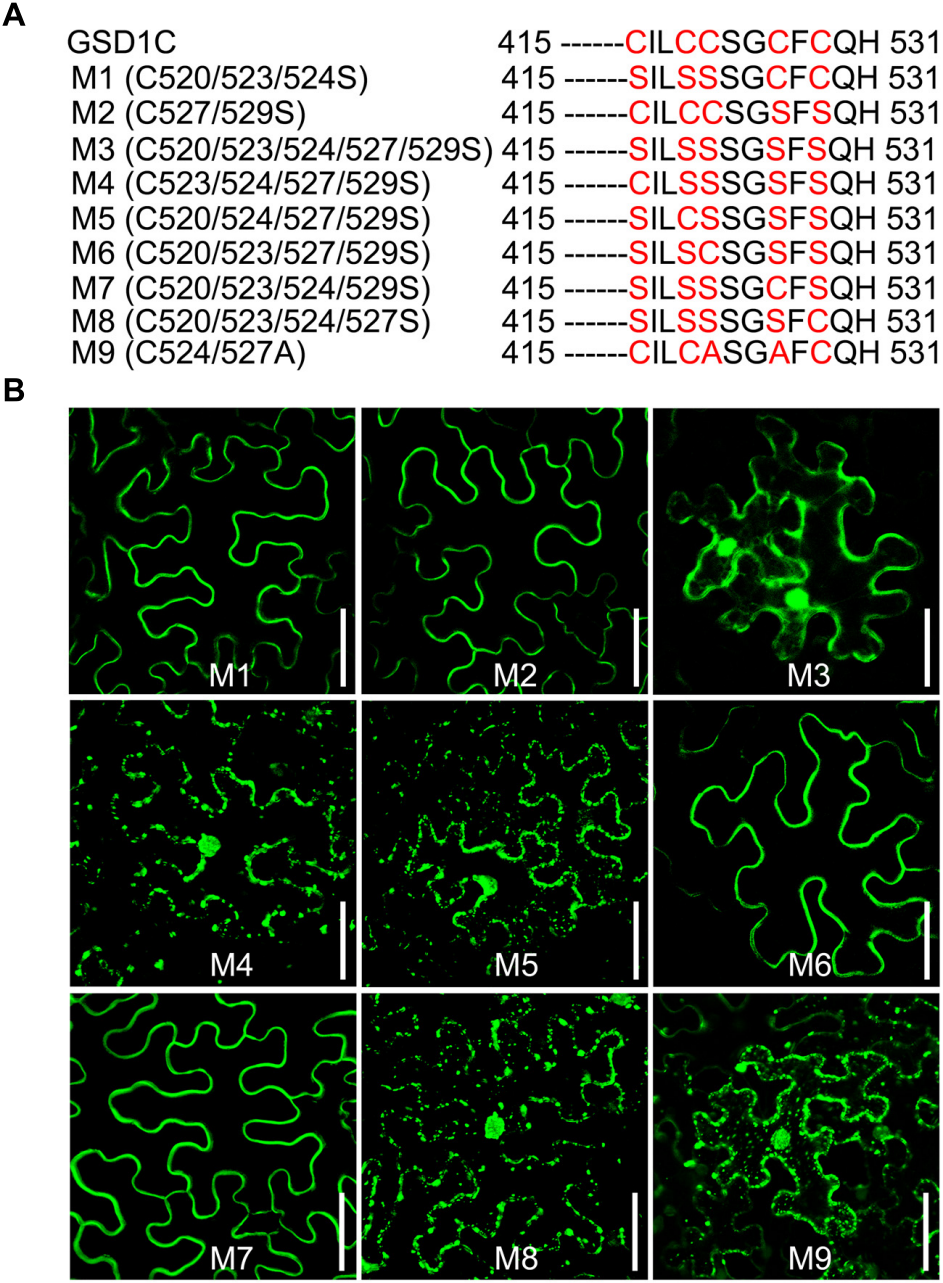
**The cysteine residues Cys-524 and Cys-527 are essential for GSD1 PM targeting and *S*-acylation. (A)** Schematic representation of multiple mutations of cysteine residues in the GSD1 C-terminal sequence. **(B)** Subcellular localization of GSD1 multiple mutations in tobacco leaves. The quadruple mutations M6 and M7 displayed a specific localization on PM. Bars = 50 μm.

Four (C523/524/527/529) of the five cysteine residues (C520/523/524/527/529) in the GSD1 C-terminal are predicted by CSS-Palm 2.0 (Ren et al., 2008) to be S-acylated (Supplementary Figure S5B). For identification of the cysteine residue *S*-acylation, mutations to cysteine to serine were carried out (**Figure 4A**, Supplementary Figure S6A). Individual mutation of each cysteine (C520, C523, C524, C527, and C529) did not change the subcellular localization of GSD1 on the PM (Supplementary Figure S6B). When multiple mutations were made (**Figure 4A**), GSD1 PM localization was not changed in triple (M1, C520/523/524 to S520/523/524) or double (M2, C527/529 to S527/529) mutants but was altered in quintuple mutants (M3, C520/523/524/527/529 to S520/523/524/527/529) in which GSD1 was localized in the cytoplasm and nucleus (**Figure 4B**). Combinational quadruple mutations (M4, C523/524/527/529 to S523/524/527/529; M5 C520/524/527/529 to S520/524/527/529; M6 C520/523/527/529 to S520/523/527/529; M7 C520/523/524/529 to S520/523/524/529; M8 C520/523/524/527 to S520/523/524/527) (**Figure 4B**) were also performed. For quadruple mutants M4, M5, and M8, GSD1 was found to be present in the cytoplasm and nucleus, whereas mutants M6 and M7 displayed a specific localization on PM, similar to that of wild type GSD1 (**Figure 4B**). For further confirmation that both Cys-524 and Cys-527 are crucial for GSD1 PM localization, we mutated Cys524/527 to Ala524/527 (M9, **Figure 4A**) which resulted in complete loss of GSD1 association with the PM (**Figure 4B**). Together, these results demonstrated that *S*-acylation of Cys-524 and Cys-527 is essential for GSD1 localization on PM.

### Coiled-Coil Domain of GSD1 Contributes to its Binding to OsACT1

**FIGURE 5:**
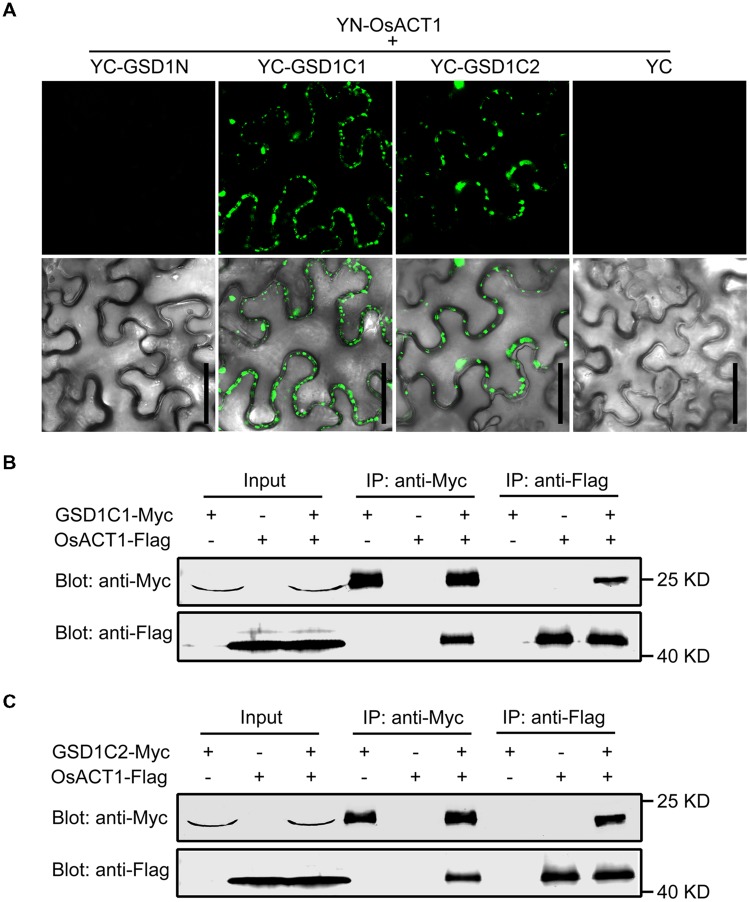
**Analysis of the GSD1 domain for binding to OsACT1. (A)** GSD1 domains (GSD1N, GSD1C1, or GSD1C2, shown in **Figure 2A**) are examined for interactions with OsACT1 using bimolecular fluorescence complementation (BiFC) assay. GSD1N: GSD1 N-terminal fragment; GSD1C1, GSD1 C-terminal fragment; GSD1C2: actin-binding domain. Bars = 50 μm. **(B)** and **(C)** Interaction of GSD1C1-Myc **(B)** or GSD1C2-Myc **(C)** with OsACT1-Flag is detected by Co-immunoprecipitation (Co-IP). GSD1-Myc and OsACT1-FLAG were individually expressed or combinationally co-expressed in tobacco leaves. Total protein extract was immunoprecipitated with anti-Myc antibodies coupled agarose beads or anti-Flag antibodies coupled agarose beads. Proteins from crude lysate and immunoprecipitation were detected with anti-Myc antibodies and anti-Flag antibodies, respectively.

We showed in a previous study that GSD1 affects the grain setting in rice through interacting with OsACT1 to regulate PD conductance. However, the part of the GSD1 structure responsible for interactions with OsACT1 is unknown. To investigate how GSD1 interacts with OsACT1, the full-length GSD1 was truncated to GSD1N (N-terminal part of GSD1), GSD1C1 (C-terminal part of GSD1) and GSD1C2 (the coiled-coil domain and adjacent residues in the C-terminal part), and each truncation was fused to YFP C-terminal sequence (YC), while the YFP N-terminal sequence (YN) moiety was fused with OsACT1. When the GSD1 truncations (YC-GSD1N, YC-GSD1C1 and YC-GSD1C2) were individually co-expressed with YN-OsACT1 in tobacco leaf cells, YC-GSD1C1 and YC-GSD1C2 were shown to interact with YN-OsACT1, however, both free YC and YC-GSD1N were not shown to interact with YN-OsACT1 (**Figure 5A**, Supplementary Figure S7A). The fluorescent interaction signal was detected predominantly on the PM with an uneven distribution (**Figure 5A**). These results indicated that the C-terminal region containing the coiled-coil domain in GSD1 acts as an actin-binding domain mediating the interaction with OsACT1. To verify the interaction of the actin-binding domain with OsACT1, the GSD1 was sectioned into GSD1N, GSD1C1, and GSD1C2 fragments used in the subcellular localization and BiFC analyses. Myc-fused GSD1 fragments (GSD1N-Myc, GSD1C1-Myc, and GSD1C2-Myc) and Flag-fused OsACT1 (OsACT1-Flag) were prepared and used for co-immunoprecipitation (Co-IP). As shown in **Figure 5B**, the anti-Myc antibody-coupled agarose beads were co-precipitated with both OsACT1-Flag and GSD1C1-Myc. Conversely, the anti-Flag antibody-coupled agarose beads were able to co-precipitate both GSD1C1-Myc and the OsACT1-Flag. In contrast, GSD1N-Myc and OsACT1-Flag did not show co-precipitation together (Supplementary Figure S7B). In addition, the anti-Flag antibody-coupled agarose beads were not able to pull down GSD1C1-Myc along with Myosin1-Flag or Myosin2-Flag, which are the cytoskeleton components localized in plasmodesmata (Supplementary Figure S7C). These results demonstrate that the C-terminal region of GSD1 mediated the specific interaction with OsACT1. In addition, the anti-Myc antibody as well as anti-Flag antibody-coupled agarose beads co-precipitated with both GSD1C2-Myc and OsACT1-Flag, further confirming the direct interaction of the GSD1 coiled-coil domain (GSD1C2) with OsACT1 (**Figure 5C**). Collectively, these results demonstrate that the coiled-coil domain is necessary and sufficient for GSD1 to specifically interact with OsACT1.

### *S*-acylation-Mediated PM Localization is Required for GSD1 Function

**FIGURE 6:**
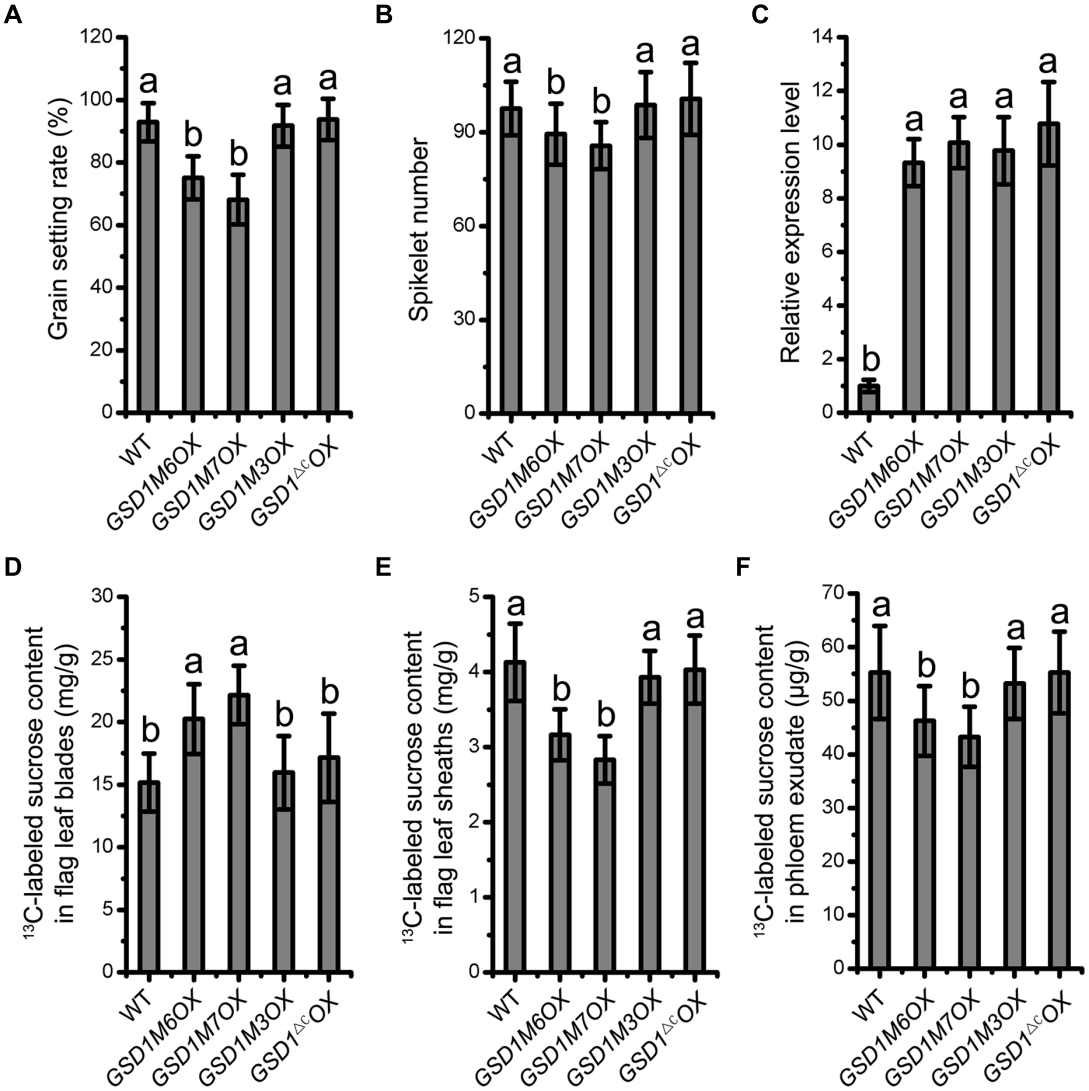
***S*-acylation is crucial for GSD1 regulation of the PD conductance. (A)** and **(B)** Statistical analyses of grain setting rate **(A)** and spikelet number **(B)** in WT, GSD1 cysteine mutants (*GSD1M3OX, GSD1M6OX, GSD1M7OX*) and GSD1 deletion mutant (*GSD1^ΔC^OX*) transgenic plants. Values are means ± SE of 15 independent plants. **(C)** Quantitative RT-PCR analyses of the GSD1 expression in panicles at flowering stage. Results are means ± SE of three individual samples. **(D–F)** Measurement of the ^13^C-labeled sucrose in flag leaf blades **(D)**, flag leaf sheaths **(E)** and phloem exudate **(F)** after flag leaf blade photosynthesis fed with ^13^CO_2_.

Deletion of the 12 C-terminal amino acid residues and mutation of the Cys-524 and Cys-527 residues in GSD1, which abolishes GSD1 *S*-acylation, results in the disassociation of GSD1 from the PM. We next investigated how the deletions and mutations affected the functionality of GSD1 in rice. Our previous study demonstrated that *GSD1* overexpression (*GSD1OX*) caused a reduction in grain setting in rice as a result of inhibiting photoassimilate transport. Four modified *GSD1* constructs (a *GSD1* deletion of the 12 amino acid sequence in the C-terminal, *GSD1^ΔC^* and the three cysteine mutants, *GSD1M3, GSD1M6*, and *GSD1M7*) were constructed and overexpressed in rice to generate *GSD1^ΔC^OX, GSD1M3OX, GSD1M6OX*, and *GSD1M7OX* transgenics. 15 independent transgenic lines from each construct were examined and results showed that *GSD1M6OX* and *GSD1M7OX* transgenic rice plants similar to *GSD1OX* exhibited obvious defects in grain setting compared with wild-type, whereas *GSD1^ΔC^OX* and *GSD1M3OX* transgenics exhibited no obvious defects in grain setting in compared with the wild-type, despite all of the transgenics displaying similar expression level (**Figures 6A,C**). Spikelet number in *GSD1M6OX* and *GSD1M7OX* transgenics were also significantly reduced as compared to the wild-type, *GSD1^ΔC^OX* or *GSD1M3OX* transgenics (**Figure 6B**).

To measure photoassimilate transport from photosynthetic leaf to phloem, ^13^CO_2_ was fed to rice leaf and stable isotopic labeled sucrose content was determined in leaf blades, leaf sheaths, and phloem exudates according to established method (Gui et al., 2014). The ^13^C-labeled sucrose in leaf blades, leaf sheaths, and phloem exudates exhibited significant difference between the transgenic plants (*GSD1M6OX* and *GSD1M7OX*) and wild-type. However, no significant difference was observed between the mutated GSD1 transgenic plants (*GSD1^ΔC^OX* and *GSD1M3OX*) and wild-type (**Figures 6D–F**). These results demonstrated that the GSD1 C-terminal two cysteines (Cys-524 and Cys-527) *S*-acylation is essential for GSD1 function.

## Discussion

Remorins are a type of plant specific proteins associated with the PM. The functional diversity of remorins can be attributed to their membrane platform association and their interaction with other proteins. In rice, 19 remorin genes with majority of unknown function show highly diverse sequences with a range of sequence identity from 13 to 39% (Gui et al., 2014). Rice *GSD1*, belonging to remorin group 6, affects grain filling through regulating the loading or unloading of photoassimilate through the phloem transport system (Gui et al., 2014). In this study, we examined the mechanistic role of GSD1 in association with the PM and its interaction with actin.

The study reveals that GSD1 is associated with the PM at the cytoplasmic face. As the GSD1 amino acid sequence displays overall hydrophilic properties, post-translational modification is required for its membrane association. A recent study indicated that many remorin proteins are associated with membrane through *S*-acylation (Konrad et al., 2014). However, no consensus motifs have been identified for protein *S*-acylation. The exact prediction of the *S*-acylation sites is rather difficult (Roth et al., 2006; Hemsley et al., 2013). There are five cysteine residues in the region predicted for GSD1 *S*-acylation. Characterization of the exact *S*-acylation sites would be essential for understanding of the GSD1 function. Deletion and mutation analyses revealed that a stretch of 45 amino acid residues at the C-terminal is a primary sequence for GSD1 anchoring on the PM. The membrane association is through *S*-acylation modification at two cysteine residues Cys-524 and Cys-527 among the five cysteine residues within the sequence. The sequence structure for *S*-acylation varies among different remorins and homologs in difference species as well. A GSD1 homolog in *Arabidopsis* (At4g36970) contains only two cysteine residues in a sequence of 35 amino acid residues at the C-terminal which is predicted and demonstrated for *S*-acylation (Konrad et al., 2014). In comparison of the remorin sequences, the S-acyaltion sequence structure of GSD1 is not conserved among remorins. Actually remorins are localized in various subcellular sites or even associated with different microdomains on the PM (Jarsch et al., 2014; Konrad et al., 2014). Such diverse membrane association can be attributed to remorin sequence structures as well as interaction with different proteins. In present study, results revealed that the rice GSD1 *S*-acylation through two cysteine residues within a stretch of 45 amino acid residues is able to secure its PM localization. The variable S-acylaton sequence structure in remorins may be associated with diverse membrane compartmentalized domains, with which remorins are localized for carrying out different functions.

Grain setting defect1 belongs to remorin group 6 and is the only group 6 remorin, whose function has been studied (Gui et al., 2014). This group of remorins contains a relatively long length of amino acid sequences with a highly variable N-terminal sequence which may be involved in protein–protein interactions (Raffaele et al., 2007). However, how different domain structures interact with other proteins to carry out their functions remain to be elucidated. Evidence from our study demonstrates that the 45 amino acid residue sequence is essential for accurate GSD1 function in regulating photoassimilate transport in rice. This suggests that the sequence structure surrounding the sites of *S*-acylation is able to localize GSD1 at correct membrane platforms and to make GSD1 function properly in rice. As the sequence surrounding the *S*-acylation sites is not conserved in remorins, the question of what sequence structures play a role in determining the localization of remorins to the right membrane compartmentalized domains remains to be addressed.

Evidence from the present study demonstrates that a new actin-binding domain at the C-terminal region of GSD1 mediates the specific and stable interaction between GSD1 and OsACT1. The results further verify our previous findings that GSD1, acting as a regulating device between desmotubule and PM, plays a role in modulating the aperture size of the PD channels. Our evidence also reveals that the C-terminal region is critical to GSD1 membrane association and interaction with actin, which is essential for GSD1 function performance in rice. How the N-terminal region is involved GSD1 function remains unclear. The N-terminal regions of the group 6 remorin show homology to xylulokinase, MurB reductase, and contain conserved MEME motifs including putative phosphorylation sites and predicted protein–protein interaction motifs (Raffaele et al., 2007). The N-terminal GSD1 region contains a putative GSK3 phosphorylation recognition site, a putative MAPK interacting motif and a predicted Tyr-based sorting signal. These motifs could be responsible for interaction with other proteins. A next study could focus on how the N-terminal region is involved in the performance of remorin function. The membrane-associated GSD1 may be localized with specific membrane platforms which involve complex interactions with a variety of partners in addition to the interactions with actin. Whether the N-terminal region plays a role in interaction with other partners has yet to be studied.

Remorins are involved in a variety of biological process, such as hormone responses, plant–microbe and plant–virus interactions. Among group 1 remorins, StREM1.3 is found to play a role in impairing potato virus × movement (Raffaele et al., 2009; Perraki et al., 2014). In *Populus deltoids*, a *PdREM* gene is reported to be involved in regulating stem development and phloem formation (Li et al., 2013). MiREM and SiREM6 are thought to be associated with defense to abiotic stress (Checker and Khurana, 2013; Yue et al., 2014). In group 2 remorin, MtSYMREM1 is found for playing a role in regulating bacterial infection by interacting with symbiotic receptors (Lefebvre et al., 2010) and LjSYMREM1, the ortholog of MtSYMREM1 in *Lotus japonicus*, is related to root nodulation (Toth et al., 2012). In the group 4, *Arabidopsis* AtREM4s play a role in regulating the cell cycle during geminivirus infection, likely through the SnRK1-mediated signaling pathway (Son et al., 2014). In the group 6, the remorin gene *GSD1* in rice has been demonstrated as a plasmodesmatal conductance regulator (Gui et al., 2014). Although several remorins have been studied for various functions in plant, understanding of the mechanisms regarding how remorins perform their diverse function is yet to be further elucidated. Mechanistic analysis of the role of GSD1 in regulating photoassimilate transport in rice revealed the sites of GSD1 *S*-acylation, the sequence essential for GSD1 localization on the PM and domain structures that facilitate its interaction with actin. These results help establish a mechanistic model to elucidate the function of remorin protein GSD1 in regulating plasmodesmata conductivity in the phloem transport system of rice.

## Author Contributions

JG, designed and performed the research, analyzed data and wrote the paper; SZ and JS, performed the research; LL, designed the research, analyzed data and wrote the paper.

## Conflict of Interest Statement

The authors declare that the research was conducted in the absence of any commercial or financial relationships that could be construed as a potential conflict of interest.
