# Supplementary material for: Grain setting defect1 (GSD1) function in rice depends on S-acylation and interacts with actin 1 (OsACT1) at its C-terminal
Source: Front Plant Sci. 2015 Oct 1;6:804. doi: 10.3389/fpls.2015.00804 (PMC4590517; doi:10.3389/fpls.2015.00804)
Supplement: Supplementary file 1 [file Table_1.PDF]

Supplemental Table 1. List of primers used in this study.

Primer name	Primer sequence (5’→3’)	Purpose
GSD1 F	TTCCCCAGTCCCTCCATCT	Cloning
GSD1 R	CGTTGAGCAACAACCAAGTGT	
AtPDLP1 F	AAACAAAAGACAAAAAAAAAACG	Cloning
AtPDLP1 R	AAAAATAAGAATCAATAAGCATCATA	
Q-GSD1 F	ACAGAAAAGGTGGCACAAAT	QRT-PCR
Q-GSD1 R	ATGGCTTCGCTTCGCAAT	
OsAct1 F	TCGTCTGCGATAATGGAACT	QRT-PCR
OsAct1 R	CTCGATGGGGTACTTGAGG	
314AtPDLP1 F	TCTCTAGAATGAAACTCACCTA	Subcellular localization
314AtPDLP1 R	AAGGATCCATAAGCATCATA	
928GSD1F	AGAGTCTAGAAATGGAGTATGAAAG	Subcellular localization and BiFC
928GSD1R	ACAAGATCTTTGACAGAAGCAAC	
GSD1N F	AATCTAGACATGGAGTATGAAAGG	Subcellular localization and BiFC
GSD1N R	TTAGATCTTTCTTTCTTCATCCG	
GSD1C1 F	AATCTAGAGAAAGAATATGCAGC	Subcellular localization and BiFC
GSD1C1 R	TTGGATCCGTGTTGACAGAAG	
GSD1C2 R	TTGGATCCTTCAGCTAAACG	Subcellular localization and BiFC
GSD1C3 F	AATCTAGAAGCTGAAGAGAAACG	
GSD1C4 F	AAAAATCTAGAAGCTGAAGAGAAACG	Subcellular localization
GSD1C4 R	AAATTGGATCCCTGGCGAATCA	
GSD1C5 F	TTTAATCTAGAGACAGGACGAGTTC	Subcellular localization
GSD1C5 R	AAATTGGATCCGTGTTGACAGA	
2012GSD1C1 F	TTTAACTCGAGATGGAATATGCAG	Subcellular localization
2012GSD1C1 R	AAATTACTAGTTTGTGTTGACAGAAG	
218GSD1C6 F	TTTAATCTAGAGAAAGAATATGCAGC	Subcellular localization
218GSD1C6 R	AATATGGATCCTGACCCTGGAACTC	
2011-YFPN F	TAGTCTCGAGATGGTGAGCAAG	BiFC
2011-YFPN R	AGCTCTAGAGCCATGATATAGACG	
2011-YFPC F	CACTCGAGATGGACAAGCAG	BiFC
2011-YFPC R	TTCTAGAGTCTTGTACAGCTCGTC	
218GSD1C F	TTTAATCTAGAGAAAGAATATGCAGCTCG TGCAGCAGC	Subcellular localization of cysteine mutagenesis
218C520S R	AAATTGGATCCGTGTTGACAGAAGCAAC CACTGCAGCATAGGATACTTGAC	
218C523S R	AAATTGGATCCGTGTTGACAGAAGCAAC CACTGCAACTTAGGATACATGAC	
218C524S R	AAATTGGATCCGTGTTGACAGAAGCAAC CACTACTGCATAGGATACATGAC	
218C527S R	AAATTGGATCCGTGTTGACAGAAACTACC ACTGCAGCATAGGATACATGAC	
218C529S R	AAATTGGATCCGTGTTGACTGAAGCAAC CACTGCAGCATAGGATACATGAC	

M1 R	AAATTGGATCCGTGTTGACAGAAGCAAC CACTACTTGATAGGATACTTGAC
M2 R	AAATTGGATCCGTGTTGACTGAAACTACC ACTGCAGCATAGGATACATGAC
M3R	AAATTGGATCCGTGTTGACTGAAACTACC ACTACTTGATAGGATACTTGAC
M4R	AAATTGGATCCGTGTTGACAGAAACTACC ACTACTTGATAGGATACTTGAC
M5R	AAATTGGATCCGTGTTGACTGAAGCAAC CACTACTTGATAGGATACTTGAC
M6R	AAATTGGATCCGTGTTGACTGAAACTACC ACTGCATGATAGGATACTTGAC
M7R	AAATTGGATCCGTGTTGACTGAAACTACC ACTACTGCATAGGATACTTGAC
M8R	AAATTGGATCCGTGTTGACTGAAACTACC ACTACTTGATAGGATACATGAC
M9R	AAATTGGATCCGTGTTGACAGAAAGCAC CACTAGCGCATAGGATACATGAC
