# Supplementary material for: Grain setting defect1 (GSD1) function in rice depends on S-acylation and interacts with actin 1 (OsACT1) at its C-terminal
Source: Front Plant Sci. 2015 Oct 1;6:804. doi: 10.3389/fpls.2015.00804 (PMC4590517; doi:10.3389/fpls.2015.00804)

Supplemental Figure Legends

Figure S1. GSD1 association with the cytoplasmic face of PM.

(A) GSD1 fused to GFP (GFP-GSD1) was expressed in rice and tobacco protoplasts. GFP-GSD1 green fluorescence is localized on PM. Images show green fluorescence, chlorophyll red fluorescence and differential interference contrast overlay. RP, rice protoplasts; TP, tobacco protoplasts. Bars = 25 µm.

(B) Western blotting detection of GSD1 in soluble and membrane fractions from GFP-GSD1 expressing rice and tobacco protoplasts. T, total; S, soluble; MF, membrane fractions.

(C) Western blotting detection of GSD1 after treatment with proteinase K in presence or absence of detergent triton X-100.

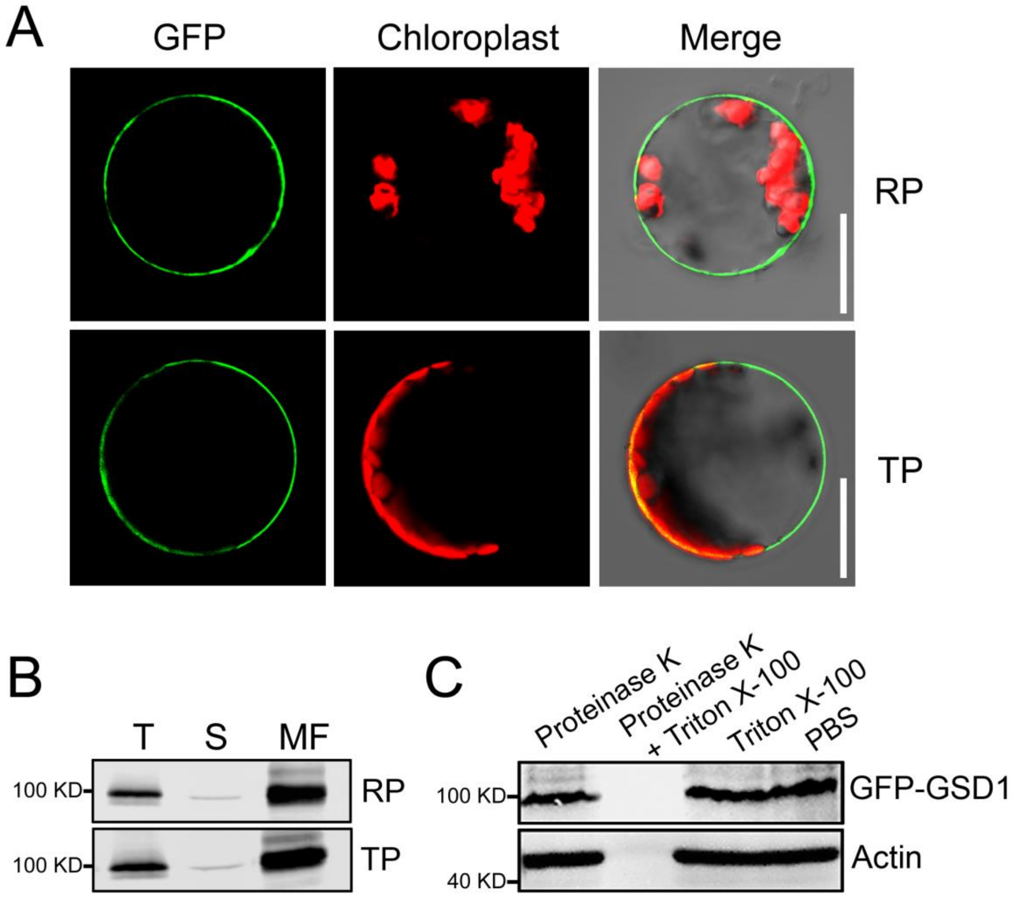

Figure S2. Domain organization and sequence alignment of GSD1.

(A) Predicted motifs or domains in Remorin_N and Remorin_C of GSD1.

(B) Domains observed in GSD1 are displayed in multiple-sequence alignment of GSD1 homologs in group 6 of rice. Black and gray denote identical and 80% conservation, respectively.

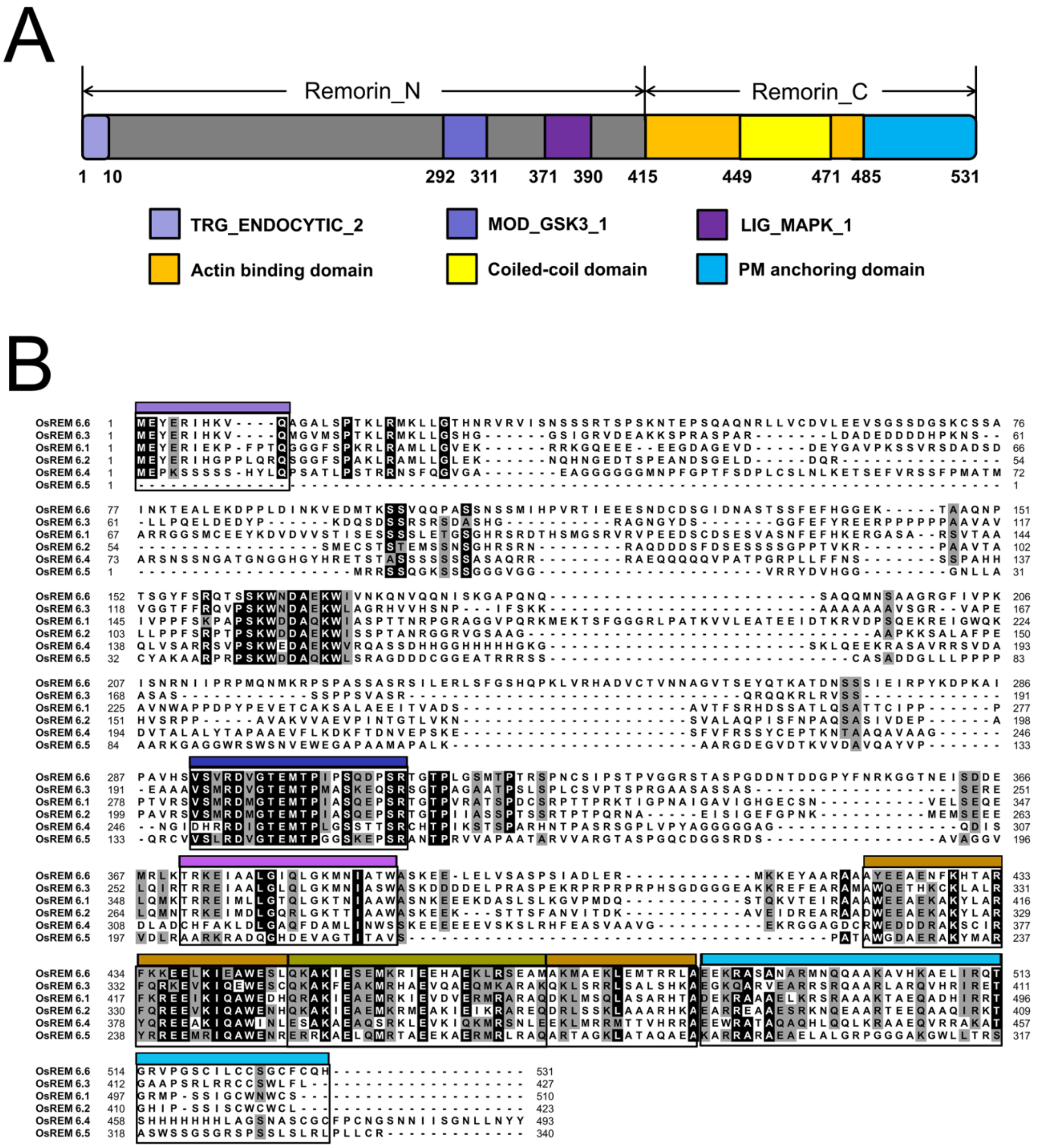

Figure S3. Plasmolysis of GFP fused GSD1 truncated mutants in N. benthamiana leaves.

After plasmolysis, GFP-GSD1C3 GFP fluorescence was detected in the PM, while GFP-GSD1C4 GFP fluorescence was detected in cytoplasm and nucleus. The GFP fluorescence of GFP-GSD1C5 was detected predominantly in the PM, but also in the nucleus. Bars = 50 μm.

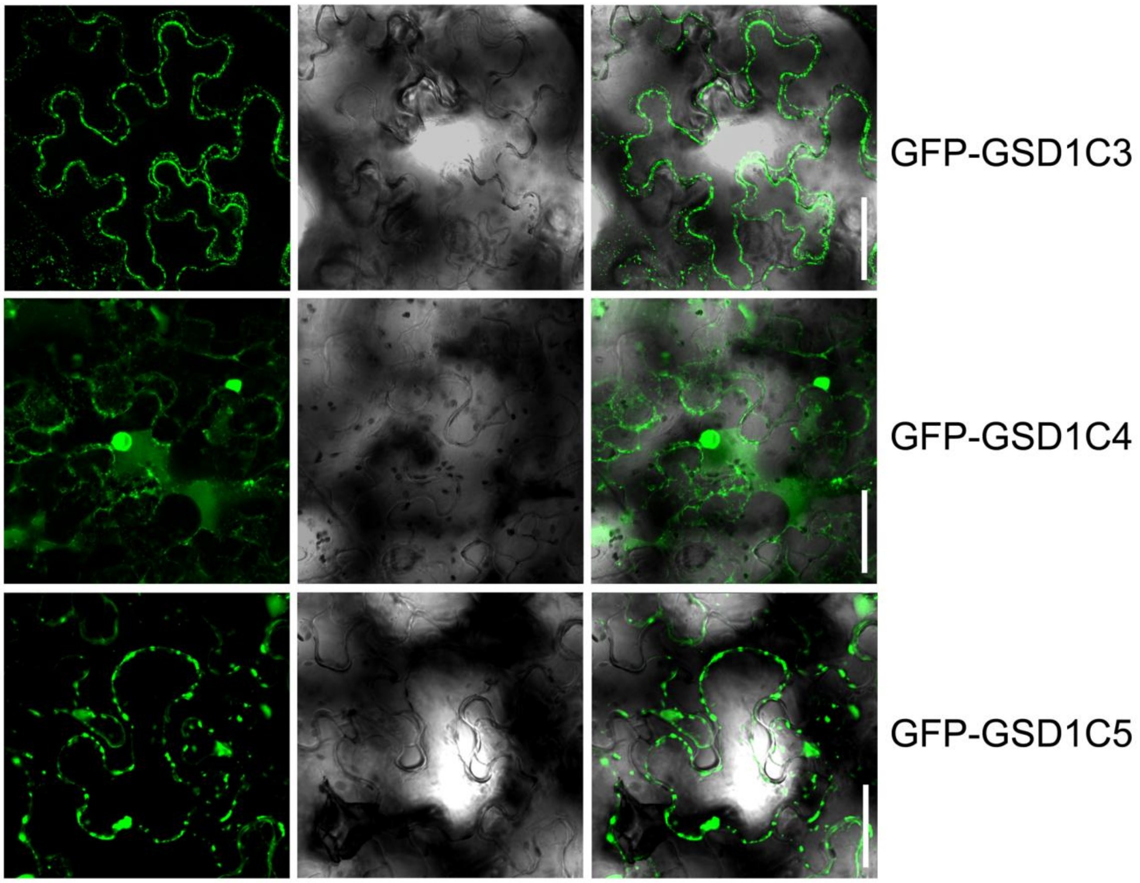

Figure S4. Trafficking of GSD1 to the PM via a novel ER/Golgi inhibitors insensitive Pathway.

Confocal images show the GFP and mCherry fused constructs were coexpressed in N. benthamiana leaves. Images show that GSD1 trafficking to PM was unaffected by BFA treatment, while ER-Golgi transport plasmadesmata protein PDLP1:mCherry plasmodesmata specific localization were completely disrupted by BFA. In addition, COPII-mediated ER-Golgi transport regulator Sar1 mutant Sar1H74L-mCherry disrupt PDLP1 plasmodesmata specific localization. Bars = 50 μm.

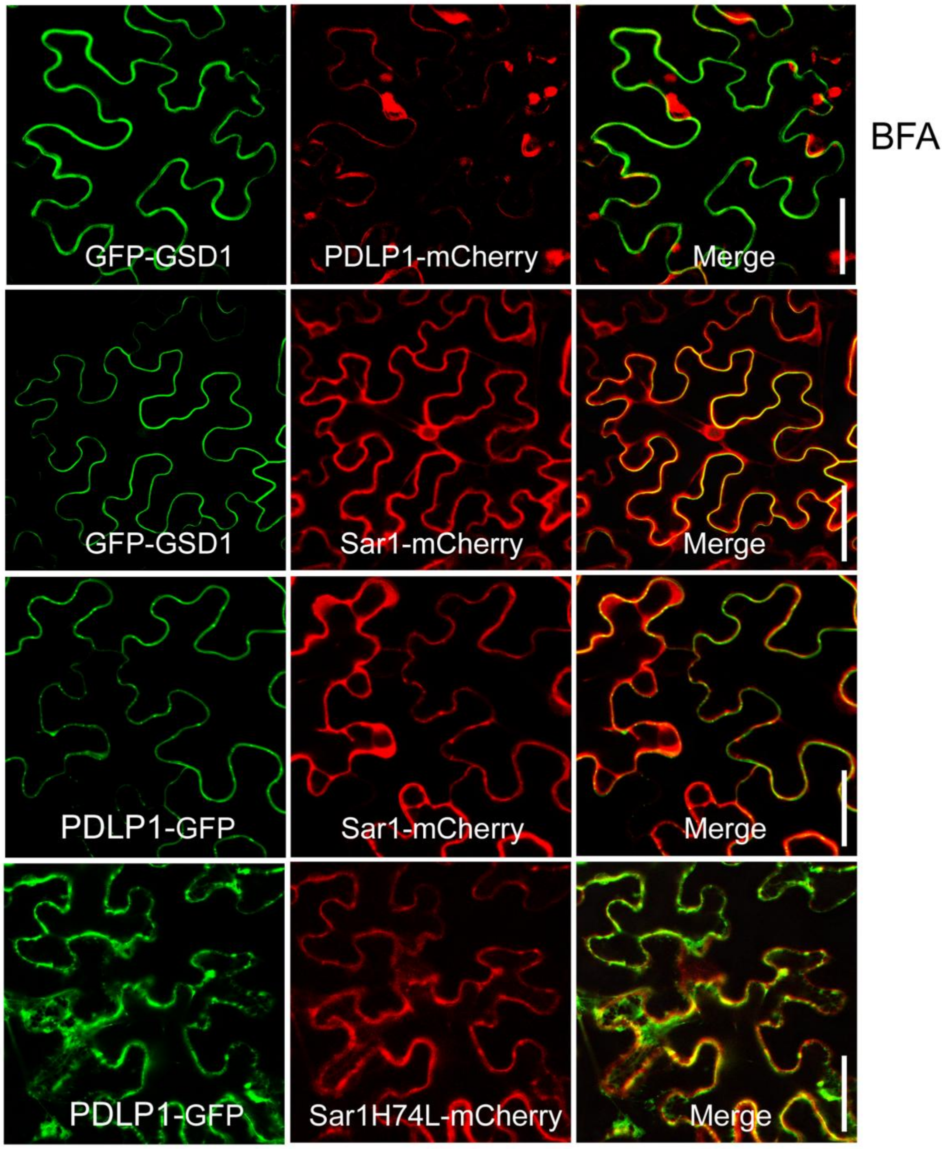

Figure S5. GSD1 protein hydrophobicity and S-acylation prediction.

(A) Prediction of hydrophobicity of the full-length GSD1 by ProtScale (Kyte and Doolittle, 1982). The prediction was based on the scale of Hphob. / Kyte & Doolittle, a linear of weight variation model, and a window of 3.

(B) Prediction of S-acylation sites in the full-length GSD1 by CSS-Palm 3.0 with high threshold (Ren et al., 2008).

A

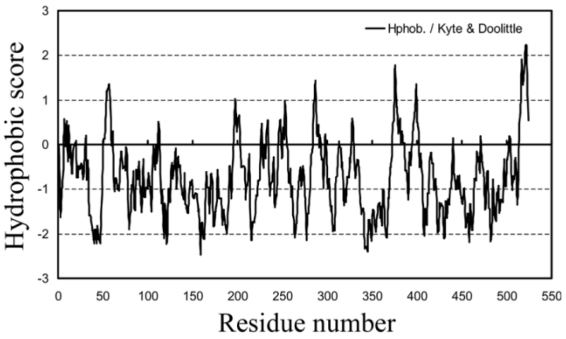

B

Position	Predicted S-acylation sites	Score
58	AQNRLLV**C**DVLEEVS	1.041
523	PGSCIL**C**CSGCFCQH	2.724
524	PGSCILC**C**SGCFCQH	2.111
527	PGSCILCCSG**C**FCQH	2.935
529	PGSCILCCSGCF**C**QH	1.843

Figure S6. Single mutagenesis of each one of the C-terminal five cysteines in GSD1 does not affect its subcellular localization.

(A) Schematic represents a series of cysteine residue point mutants in the C-terminal domain of GSD1.

(B) Confocal microscopic observation the subcellular localization of GSD1 point mutants in N. benthamiana leaves. Bars = 50 μm.

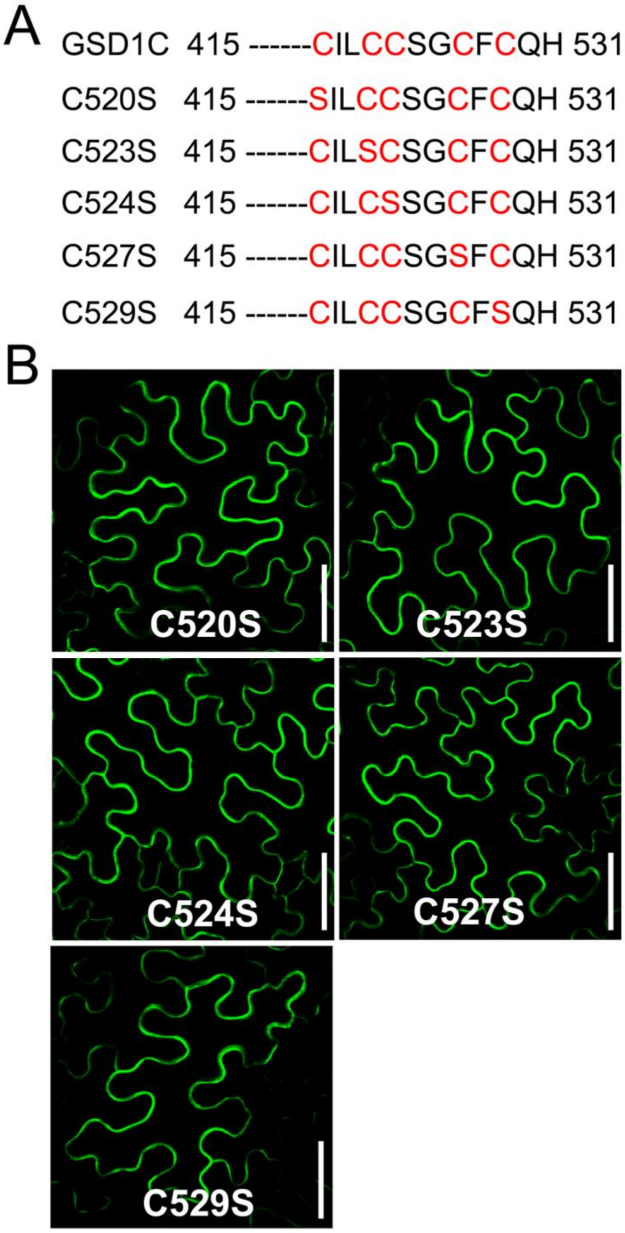

Figure S7. GSD1 specific interacts with OsACT1.

(A) Western blot detection of YC-GSD1N and YN-OsACT1 co-expression in tobacco leaves using GSD1 and actin specific antibodies, respectively.

(B) Interaction between GSD1N-Myc and OsACT1-Flag was examined in tobacco leaves by Co-immunoprecipitation (Co-IP). Total protein extracts were immunoprecipitated with anti-Myc antibody coupled agarose beads or anti-Flag antibody coupled agarose beads, respectively. Then the proteins from crude lysates and immunoprecipitated proteins were detected with anti-Myc antibodies and anti-Flag antibodies, respectively. Results show that OsACT1 was not co-precipitated with GSD1N.

(C) Interaction of OsACT1, Myosin1 (Os07g37560) or Myosin2 (Os10g34710) with GSD1C1 was examined in tobacco leaves by Co-IP. Proteins from the crude lysates and immunoprecipitated proteins were detected with anti-Myc antibodies. Results show that OsACT1 is co-precipitated with GSD1, whereas Myosin1 and Myosin1 could not co-precipitate with GSD1.

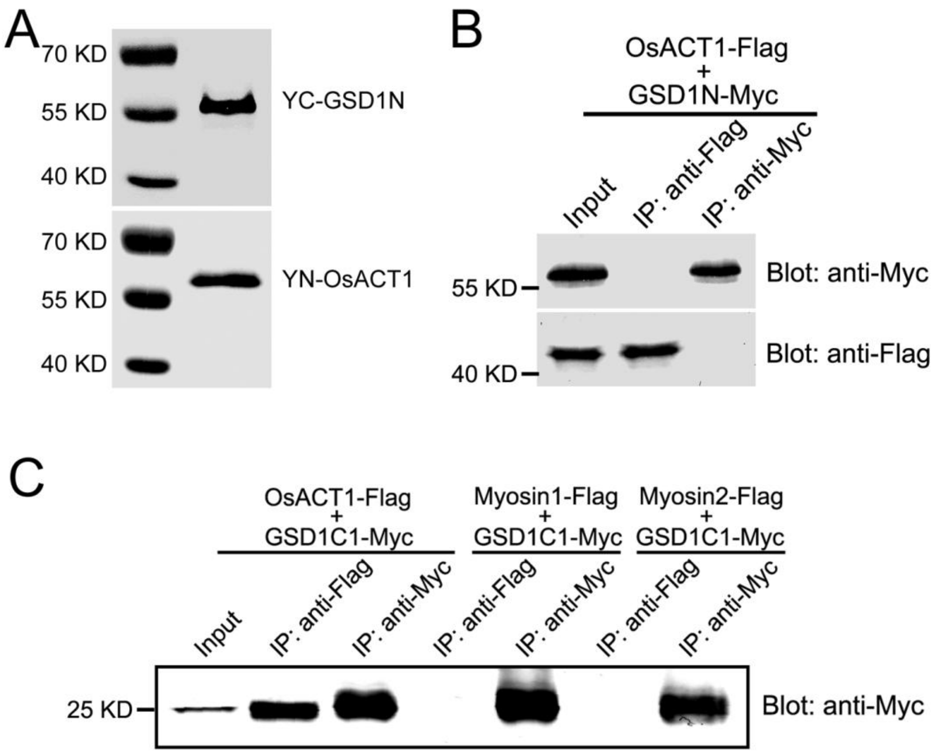

Supplement: Supplementary file 2 [file Image_1.PDF]
